# VPS37A loss creates CASP8-dependent vulnerability via the MAP3K7–NF-κB–CFLAR axis

**DOI:** 10.1038/s41417-026-01054-3

**Published:** 2026-07-07

**Authors:** Tatsuya Hattori, Longgui Chen, Xinwen Liang, Kouta Hamamoto, Hong-Gang Wang, Yoshinori Takahashi

**Affiliations:** 1 Division of Pediatric Hematology and Oncology, Department of Pediatrics, The Pennsylvania State University College of Medicine, Hershey, PA 17033 USA; 2 Department of Cell and Biological Systems, The Pennsylvania State University College of Medicine, Hershey, PA 17033 USA

**Keywords:** Cell biology, Cancer

## Abstract

VPS37A, a subunit of ESCRT-I involved in endosomal sorting and autophagy, is frequently downregulated in diverse human cancers. In this study, we showed that VPS37A downregulation, as part of a large chromosome 8p deletion, arises early during tumorigenesis and persists throughout tumor progression. Integrative analysis of VPS37A gene copy number and CRISPR dependency revealed that VPS37A deficiency creates a synthetic lethal dependency on the MAP3K7–NF-κB–CFLAR axis, and targeting this axis triggered CASP8-mediated apoptosis and suppressed tumor growth. This synthetic vulnerability depends on ATG8ylated membranes, which serve as a platform for CASP8 activation upon inhibition of phagophore closure, and can be triggered without disrupting receptor sorting. Consistently, despite frequent co-deletion of VPS37A and the death receptors TNFRSF10A/B, inhibition of the MAP3K7–NF-κB–CFLAR axis selectively induced apoptosis in spheroid tumors with 8p deletion. These results uncover a selective vulnerability in cancer cells harboring VPS37A/8p loss, providing a mechanistic rationale for targeted therapeutic intervention.

## Introduction

Macroautophagy (hereafter autophagy) is a catabolic process in which cytoplasmic constituents are sequestered and delivered to lysosomes for degradation and nutrient recycling [1]. Autophagy plays vital roles in protein and organelle quality control, as well as in cellular adaptation to nutrient limitation during starvation. In cancer, impaired autophagy promotes tumor initiation by allowing the upregulation of reactive oxygen species, leading to genomic instability. Conversely, in established tumors, inhibition of autophagy-mediated stress adaptation impairs tumor progression and metastasis [2, 3]. Thus, through its roles in degradation and nutrient recycling, autophagy exerts paradoxical functions, suppressing tumor initiation while promoting tumor progression.

A central event in autophagy is the formation of a crescent-shaped membranous structure, the phagophore, on which the ATG conjugation machinery catalyzes covalent attachment of autophagy-related 8 (ATG8)/microtubule-associated protein 1 light chain (LC3) family proteins to phosphatidylethanolamine (ATG8ylation) [4–6]. Membrane-conjugated ATG8/LC3 proteins mediate cargo recognition and recruitment either by directly engaging LC3-interacting region (LIR)-containing substrates or indirectly via cargo receptors that contain LIRs. Additionally, ATG8ylation promotes phagophore expansion by recruiting LIR-containing ATG proteins. As the phagophore expands, its rim ultimately seals to enclose the cargoes, resulting in the formation of double-membrane autophagosomes. This process is mediated by the endosomal sorting complexes required for transport (ESCRT) machinery, which drives membrane scission to separate the inner and outer autophagosomal membranes [7, 8]. Inhibition of ESCRT-mediated phagophore closure not only impairs autophagic degradation but also leads to the accumulation of the apoptosis initiator caspase CASP8 [9], which is targeted for degradation along with other cargo molecules during autophagy [10]. While closure inhibition itself does not trigger apoptosis, it promotes CASP8 assembly on the membrane, thereby priming cells to respond to ER stress or other stimuli that activate the mitochondrial amplification loop, ultimately leading to full CASP8 activation and cell death [11]. Thus, beyond their role in cargo sequestration for degradation, phagophores can also serve as signaling platforms for the activation of cell death pathways.

VPS37A, a member of the VPS37 protein family, assembles with TSG101, VPS28, and a UMA protein (UBAP1 or MVB12A/B) into the tetrameric ESCRT-I complex, which functions in recruiting and assembling the downstream ESCRT components for membrane abscission [12]. Although VPS37A shares functional redundancy with other VPS37 paralogues (VPS37B-D) in the endosomal pathway [13, 14], recent studies highlight its indispensable role in phagophore closure [15, 16]. This unique requirement depends on its N-terminal region, which comprises a ubiquitin E2 variant-like (UEVL) domain flanked by membrane curvature-sensing motifs absent in other VPS37 homologs [17]. Mice deficient in VPS37A UEVL develop autophagy-defective phenotypes without showing apparent defects in endosomal sorting [18], further supporting the critical role of VPS37A in autophagy. Notably, while autophagy plays important roles in tumor growth [2, 3], the VPS37A gene is located on chromosome 8p, a region frequently deleted in various human cancers [19–21]. Moreover, VPS37A downregulation has been observed across multiple cancer types and is associated with poor patient prognosis [22–29]. Despite its high clinical relevance, how VPS37A-deficient cancer cells persist and the vulnerabilities they present remain unclear.

Here, we report the identification of the MAP3K7–NF-κB–CFLAR axis as a synthetic liability in VPS37A-deficient cancer cells. Targeting this pathway, either through genetic depletion of CFLAR or pharmacological inhibition of MAP3K7 and NF-κB, triggers the activation of CASP8, leading to apoptosis. Dependency on the MAP3K7–NF-κB–CFLAR axis is also observed in 8p-deleted human cancer cell lines. Consequently, targeting this axis markedly impairs the growth of VPS37A-deficient and 8p-deleted tumors. Our results reveal a vulnerability in VPS37A/8p-deleted tumors that could be exploited for precision-targeted therapy.

## Results

### Recurrent VPS37A deletion is detected early in tumorigenesis and persists across tumor progression

**Fig. 1:**
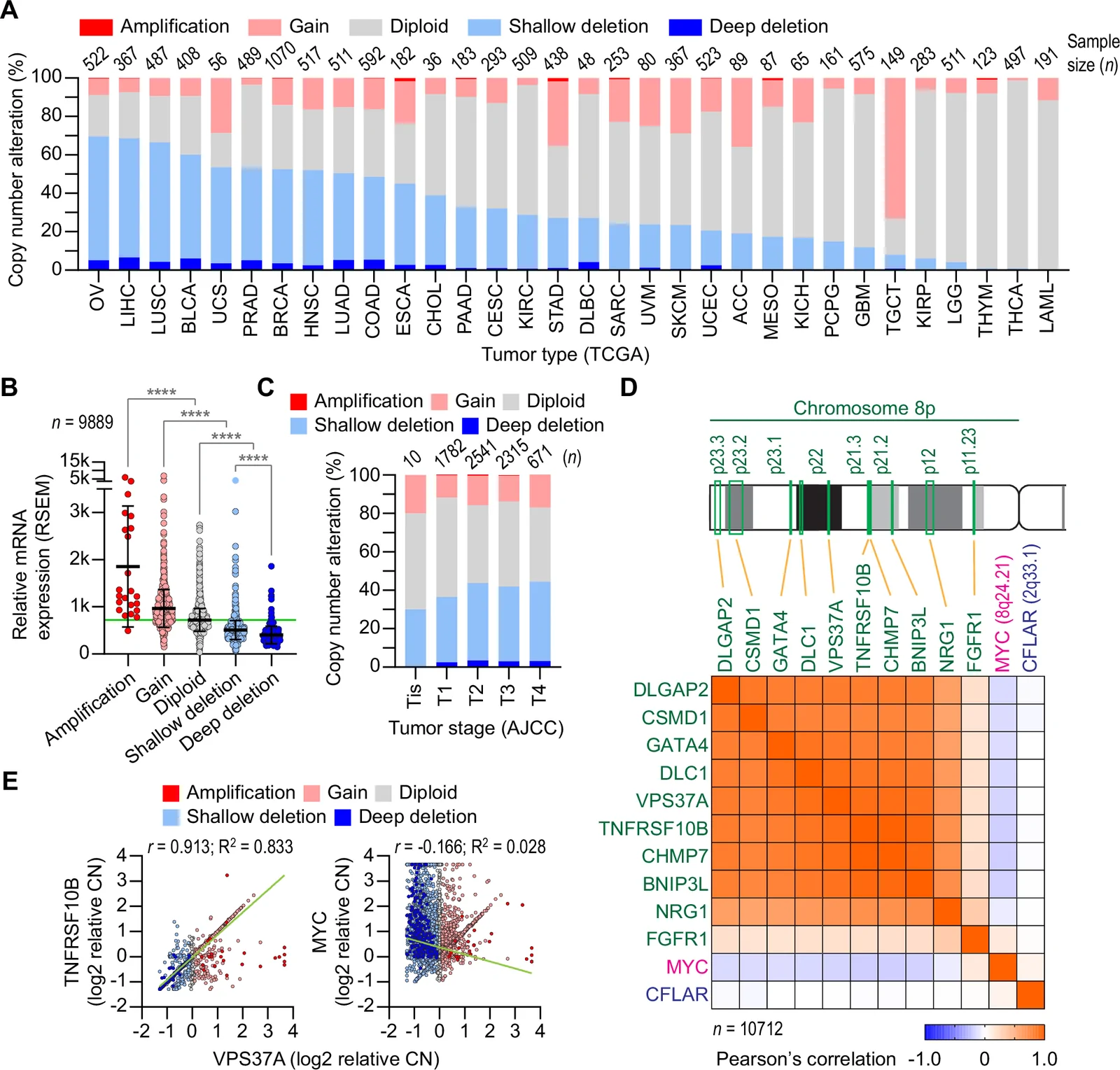
Recurrent deletion of VPS37A within large 8p chromosomal losses is present across cancer stages. **A** Bar graph showing the distribution of VPS37A gene copy number status across various tumor types in TCGA. Abbreviations for TCGA tumor types are listed in Table S1. **B** Dot plot showing the correlation between VPS37A mRNA expression levels (RSEM) and VPS37A copy number status. Statistical significance was determined by one-way ANOVA followed by Tukey’s multiple comparisons test, with pairwise comparisons performed between all groups. Individual data points are shown as dots; values are presented as mean ± SD. ****, *P* ≤ 0.0001. **C** Bar graph showing the distribution of VPS37A gene copy number status across various tumor stages in TCGA. Tis, non-invasive tumor in situ; T1–T4, tumor stages 1–4. **D** Heat map showing the correlation of copy number statuses for nine selected 8p genes (8p23.3–p11.23), along with MYC (8q24.21) and CFLAR (2q33.1). Chromosome locations of the 8p genes are displayed above. **E** Scatter plots of log₂-normalized relative copy numbers for VPS37A versus TNFRSF10B (left) or MYC (right). Simple linear regression fits are shown in green. The Pearson correlation coefficient (r) and the coefficient of determination (R²) are displayed on each plot.

Given the reported downregulation of VPS37A and the frequent deletion of chromosome 8p in multiple cancers, we analyzed The Cancer Genome Atlas (TCGA) datasets to assess the prevalence of VPS37A loss in human cancers. As expected, a high frequency of VPS37A deletion was detected across various cancer types, with the highest rates in ovarian cancer (OV, 69.6%) and liver hepatocellular carcinoma (LIHC, 68.7%), followed by lung squamous cell carcinoma (LUSC, 66.5%), bladder carcinoma (BLCA, 60%), uterine carcinosarcoma (UCS, 53.6%), prostate adenocarcinoma (PRAD, 53.2%), breast invasive carcinoma (BRCA, 52.4%), head and neck squamous cell carcinoma (HNSC, 52.0%), lung adenocarcinoma (LUAD, 50.4%), colorectal adenocarcinoma (COAD, 48.6%), and esophageal adenocarcinoma (ESCA, 45.1%) (Fig. 1A, Table S1). Approximately 90% of the deletions were shallow, indicating that most represent allelic loss rather than complete loss of VPS37A. In contrast, VPS37A loss was rarely detected in acute myeloid leukemia (LAML), thyroid carcinoma (THCA), and thymoma (THYM). We also observed VPS37A gain, most prominently in testicular germ cell tumors (TGCT, 60%). The status of VPS37A loss correlated with its expression, confirming that gene deletion underlies VPS37A downregulation in cancers (Fig. 1B). Interestingly, VPS37A deletion was already present in tumor in situ (Tis, 30.0%) and showed only a slight increase during tumor progression (T1, 36.4%; T2, 43.7%; T3, 41.9%; T4, 44.4%), suggesting that VPS37A deletion occurs at an early stage of tumorigenesis and is maintained throughout cancer development (Fig. 1C). The observed VPS37A deletion was part of a large chromosome 8p deletion, as its loss was well correlated with the loss of genes located on chromosome 8p12–p23.3 (Fig. 1D, E). Notably, VPS37A loss was frequently accompanied by an increased MYC copy number on chromosome 8q, consistent with previous reports that 8p loss and 8q gain often co-occur in human cancers [19, 20, 30]. Collectively, our analysis shows that recurrent downregulation of VPS37A, as part of a large chromosome 8p deletion, represents an early and sustained event across tumor development.

### VPS37A loss creates a CASP8-dependent synthetic vulnerability via the MAP3K7–NF-κB–CFLAR axis

**Fig. 2:**
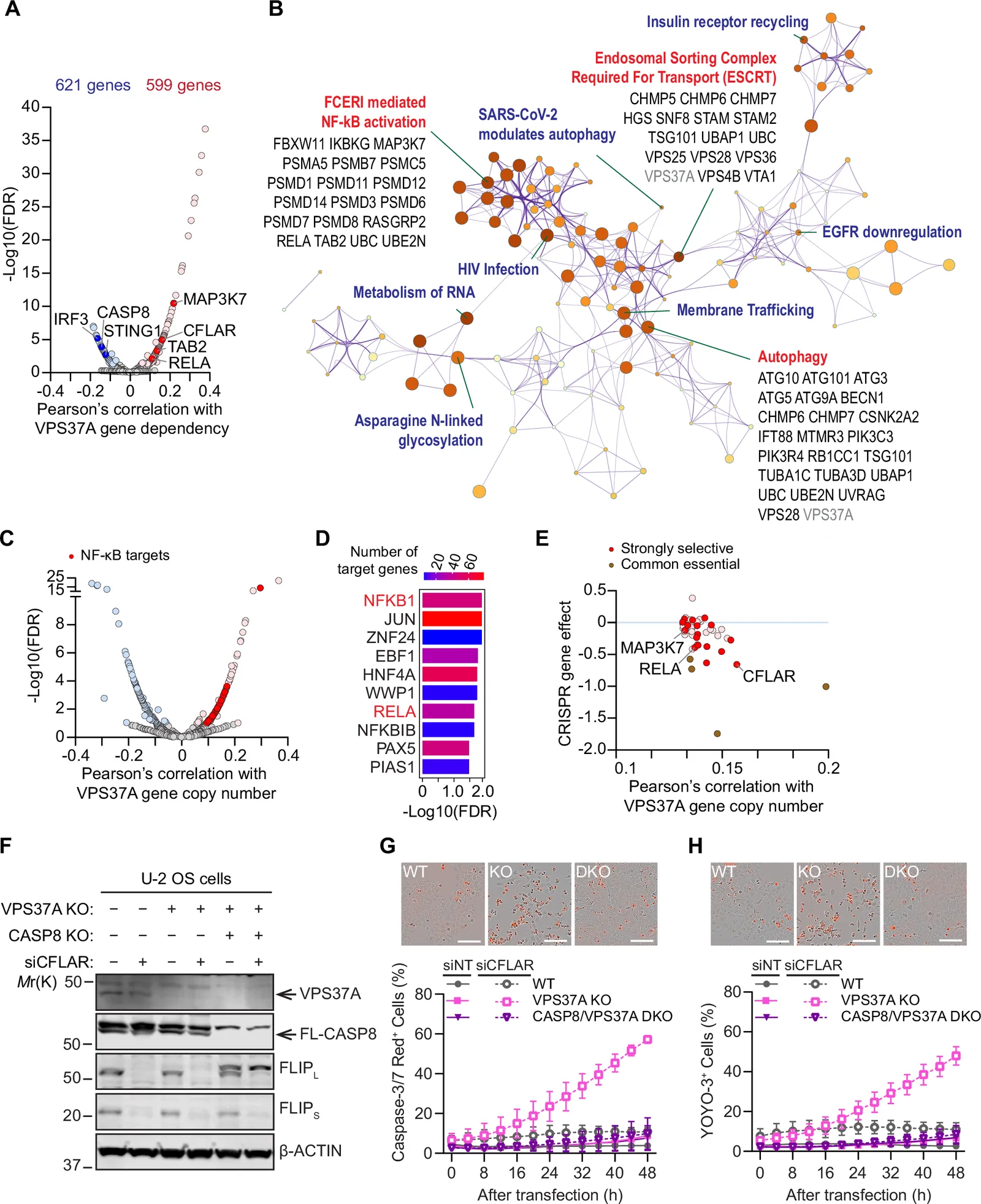
CFLAR is a synthetic vulnerability in VPS37A-deficient cells. **A** Volcano plot of genes whose CRISPR dependencies correlate with VPS37A dependency across 1183 cancer cell lines from the DepMap portal. Significant dependencies are colored (correlation > 0.1, FDR < 5%). **B** Reactome enrichment map of genes positively correlated with VPS37A co-dependency in **A**. **C** Volcano plot showing the Pearson correlation between log₂-transformed VPS37A gene copy number and CRISPR-Cas9 gene dependency across 827 cancer cell lines from the DepMap portal. Significant dependencies are colored (correlation > 0.1, FDR < 5%). NF-κB (NFKB1/REL) target genes are shown in red. **D** Bar plot of top 10 transcription factors predicted to drive the expression of the VPS37A synthetic lethal partner candidates identified in **C**. (**E**) Dot plot showing the Pearson correlation between log₂-transformed VPS37A gene copy number and the dependency of NF-κB target genes in **D**. Strongly selective and common essential genes are shown in red and tan, respectively. **F** Immunoblot analysis of indicated U-2 OS cells transfected with siNon-Targeting (siNT) or siCFLAR for 24 h. **G**, **H** Time-lapse monitoring of active CASP3/7-positive **G** and YOYO-3 iodide-positive dead **H** wild-type (WT), VPS37A knockout (KO) and CASP8/VPS37A double-knockout (DKO) U-2 OS cells after transfection with the indicated siRNA. Representative images at the 48 h-time point after siCFLAR transfection are shown on the top. Scale bar represents 150 μm. All values in the graphs are mean ± SD (*n* = 3 from three independent experiments).

To identify potential vulnerabilities in VPS37A-deficient cancer cells, we first performed a co-dependent gene analysis using The Cancer Dependency Map (DepMap) portal. We identified 1220 genes that exhibit positive or negative co-dependency with VPS37A in regulating cancer cell proliferation (FDR < 0.05) (Fig. 2A). Pathway analysis of the 599 positively correlated genes revealed ESCRT, autophagy, and NF-κB activation as the top enriched pathways (Fig. 2B, Table S2), consistent with the role of VPS37A in regulating ESCRT-mediated phagophore closure and NF-κB signaling [11, 31]. Moreover, while VPS37A loss primes cells for apoptosis by stabilizing CASP8 assembly on the phagophore [11], we observed a negative correlation between VPS37A and CASP8 CRISPR dependency (Fig. 2A), suggesting that VPS37A and the NF-κB pathway function together to limit CASP8 activation and promote cancer cell survival. Negative correlations were also detected with STING1 and its downstream effector IRF3.

To corroborate these findings, we next analyzed VPS37A copy number and CRISPR dependency data across cancer cell lines. After correlation of log2-transformed VPS37A copy number with normalized gene-level CRISPR dependency scores, 3.6% (649 genes) and 4.4% (787 genes) of the total genes (17,916 genes) showed synthetic lethal (positive correlation between CRISPR sensitivity and VPS37A gene copy number) and negative genetic interactions with the VPS37A gene, respectively, at an FDR of 5% (Fig. 2C). Among the 649 potential synthetic lethal partners, 52 genes (8%) were targets of NFKB1 and/or REL (Fig. 2C, Table S3), including 4 common essential (MAT2A, PPP2CA, RAC1, SNIP1) and 17 strongly selective genes (ACTB, CASKIN2, CFLAR, CSN2, EGFR, ESR1, ID1, KLF5, MAP3K7, MYLK2, PFKP, RELA, RXRA, SYT12, UBE2H, WWTR1, ZBTB7B) (Fig. 2D). Notably, 8 of the strongly selective genes (CFLAR, EGFR, KLF5, MAP3K7, RELA, UBE2H, WWTR1) were also identified as co-dependent with VPS37A (Fig. 2E). These data align with our previous finding that VPS37A loss confers sensitivity to MAP3K7 and IKK inhibitors [11] and support the notion that VPS37A-deficient cells are kept in check by NF-κB signaling. The dependency of VPS37A-deficient cells on NF-κB activity was further verified using JSH-23 (Fig. S1), which inhibits the nuclear translocation of the NF-κB subunit RELA/p65 [32]. In contrast, no correlation was observed between VPS37A copy number and STING1 or IRF3. Given that CFLAR, which encodes cFLIP proteins including cFLIP_L_ and cFLIP_S_ [33], is a well-established CASP8 inhibitor downstream of the MAP3K7–NF-κB axis [34] and that VPS37A loss leads to the accumulation of proCASP8 dimers on phagophores [11], we proceeded to investigate whether targeting CFLAR could selectively induce cell death in VPS37A-deficient cells. We found that siRNA-mediated CFLAR downregulation induces CASP8-dependent CASP3/7 activation and cell death only in VPS37A KO, but not in WT, U-2 OS human osteosarcoma cells (Fig. 2F–H). Collectively, we identified a CASP8-dependent synthetic vulnerability in VPS37A-deficient cells that can be exploited via inhibition of the MAP3K7–NF-κB–CFLAR axis.

### Phagophore-dependent CASP8 assembly mediates CFLAR depletion sensitivity in VPS37A-deficient cells

**Fig. 3:**
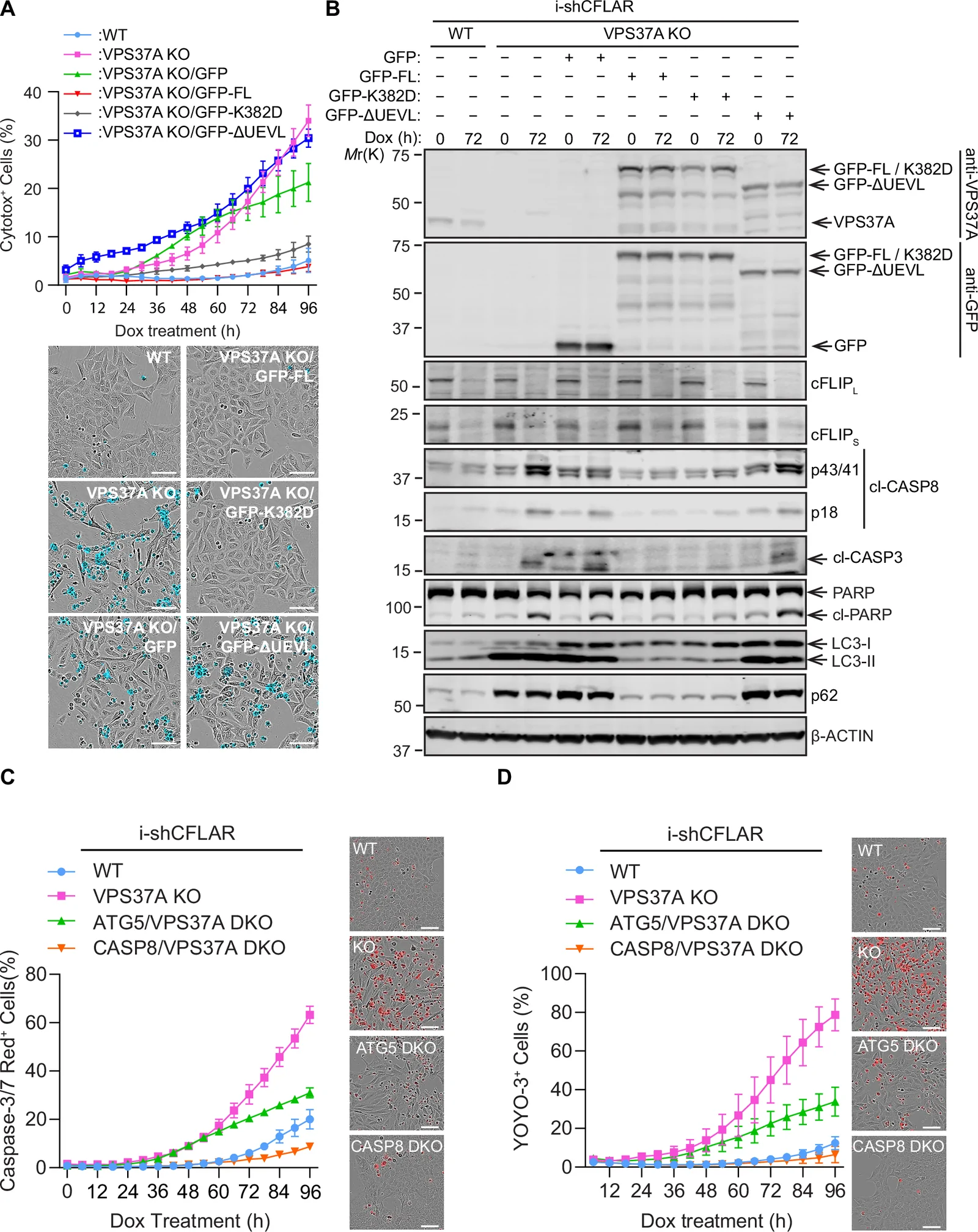
Autophagy defect, rather than endosomal sorting dysfunction, drives the sensitivity of CFLAR depletion in VPS37A-deficient cells. **A** Time-lapse monitoring of Cytotox-positive dead U-2 OS-X cells expressing inducible CFLAR shRNA (i-shCFLAR) after 1 µg/ml Dox treatment. Representative images at 96 h after Dox treatment are shown below. Scale bar represents 150 μm. **B** Immunoblot analysis of cells treated with Dox for the indicated durations. **C**, **D** Time-lapse monitoring of active CASP3/7-positive **C** and YOYO-3 iodide-positive dead **D** cells following treatment with Dox. Representative images at 96 h after Dox treatment are shown on the right. Scale bar represents 150 μm. All values in the graphs are mean ± SD (*n* = 3 from three independent experiments).

To understand the mechanism underlying the CASP8-dependent synthetic vulnerability in VPS37A-deficient cells and to assess the impact of disrupting this pathway in vivo, we generated a transplantable U-2 OS subline (termed U-2 OS-X) through in vivo passaging (see Methods). VPS37A knockout U-2 OS-X cells were established by transient transfection with VPS37A gRNA and Cas9. In addition, doxycycline-inducible shCFLAR cell lines were generated in both WT and VPS37A knockout U-2 OS-X cells to enable controlled CFLAR depletion. Consistent with parental U-2 OS cells, VPS37A loss sensitized U-2 OS-X cells to both NF-κB inhibition (Fig. S2A) and CFLAR depletion (Fig. S2B). The i-shCFLAR/VPS37A KO cells were then transduced with GFP-VPS37A full-length wild-type (FL), K382D mutant, Δ1–90 (ΔUEVL) mutant, or GFP-only control. The K382D mutation disrupts UMA protein incorporation, including the endosome-specific UBAP1, without affecting autophagy, while the UEVL deletion disrupts autophagosome closure but preserves tetrameric ESCRT-I function in endosomal receptor sorting [15, 18]. These mutants therefore enable us to dissect the distinct roles of VPS37A-containing ESCRT-I in autophagy and endosomal sorting. We found that CFLAR depletion–induced cell death in VPS37A-deficient cells was rescued not only by VPS37A full-length (FL) but also by the K382D mutant, whereas no such rescue was observed with the ΔUEVL mutant or GFP-only control (Fig. 3A). The cell death was accompanied by cleavage of CASP8 and CASP3, as well as the CASP3 substrate PARP, and correlated with the accumulation of the ATG8ylated phagophore marker LC3-II and the autophagy receptor p62 (Fig. 3B), suggesting involvement of non-canonical CASP8 activation on the phagophore. To test this, we examined the effect of disrupting ATG8ylation by knocking out ATG5, a key component of the ATG conjugation system [35]. As shown in Fig. 3C and D, CFLAR depletion-induced CASP3/7 activation and death in VPS37A KO cells were suppressed by depleting ATG5. Notably, the inhibitory effect of ATG5 loss was less robust than that of CASP8 loss, in line with previous findings that ESCRT inhibition can also enhance the canonical, death receptor–mediated apoptotic pathway [9]. Collectively, our results demonstrate that phagophore-dependent CASP8 assembly is the primary driver of apoptosis in VPS37A-deficient cells upon CFLAR depletion.

### Targeting CFLAR selectively suppresses VPS37A-deficient tumor growth

**Fig. 4:**
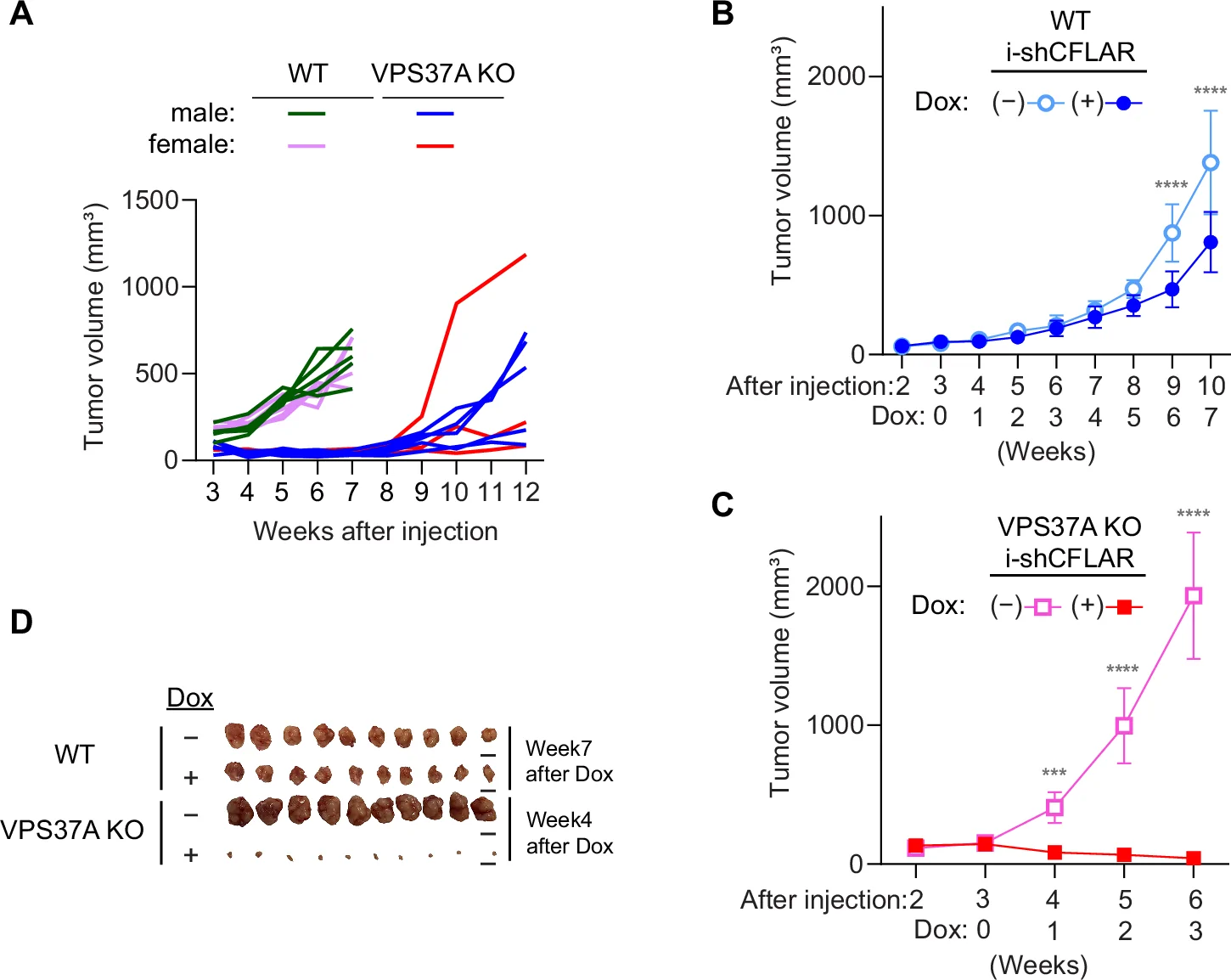
CFLAR depletion markedly suppresses growth of tumors with VPS37A loss. **A** Tumor growth curves of WT (*n* = 10) and VPS37A KO (*n* = 8) U-2 OS-X xenografts in mice. **B**, **C** Growth curves of i-shCFLAR-transduced WT U-2 OS-X **B** and VPS37A KO U-2 OS-X1 **C** xenografts in mice on Dox-containing or control diets. Group sizes were *n* = 10 for each treatment group. Data are shown as mean ± SD. ***p ≤ 0.001; ****p ≤ 0.0001. Statistical significance was determined using two-way repeated measures ANOVA followed by Tukey’s multiple-comparisons test. **D** Representative images of tumors dissected at the study endpoint (7 weeks for WT and 4 weeks for VPS37A KO) under control or Dox-containing diets. Scale bar represents 1 cm.

To evaluate the synthetic vulnerability of VPS37A-deficient cells to CFLAR depletion in vivo, i-shCFLAR/VPS37A KO and control i-shCFLAR/WT U-2 OS-X cells generated above were orthotopically injected into the tibia of NRG mice. Unlike transient depletion of VPS37A [22], tumor growth was not enhanced but instead markedly suppressed by VPS37A loss in both male and female mice (Fig. 4A), suggesting the importance of VPS37A-dependent autophagy in tumor growth. Nevertheless, approximately half of VPS37A-deficient cells eventually grew and formed tumors. Given that VPS37A downregulation is frequently observed in various tumor types [22–29], these cells were re-injected paratibially into mice. We observed that VPS37A KO U-2 OS-X1 cells acquired the ability to form tumors more rapidly than WT U-2 OS-X cells (Fig. 4B), thus allowing us to evaluate the impact of CFLAR depletion on tumor growth. Upon Dox-induced CFLAR knockdown, tumor growth was markedly suppressed in VPS37A KO U-2 OS-X1 tumors, while WT U-2 OS-X tumors showed only a modest response (Fig. 4B–D), demonstrating that VPS37A-deficient tumors retain a synthetic vulnerability to CFLAR inhibition in vivo. These results highlight the therapeutic potential of exploiting the CASP8-dependent synthetic vulnerability in VPS37A-deficient cancers.

### Cancer cells with 8p loss exhibit increased CFLAR dependency

**Fig. 5:**
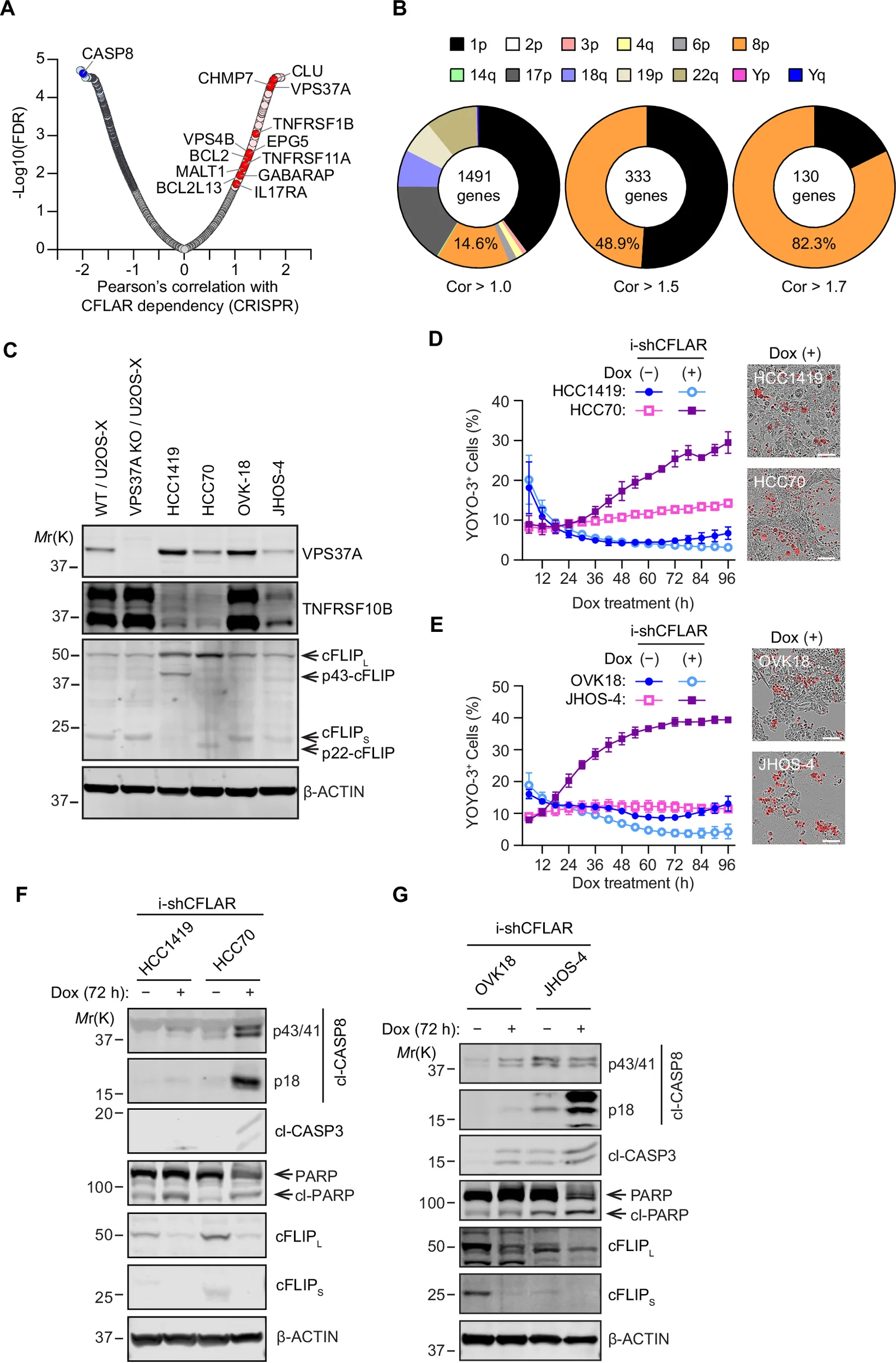
Selective vulnerability to CFLAR loss can be exploited in 8p-deleted cancer cells. **A** Volcano plot showing the Pearson correlation between CFLAR CRISPR dependency and log₂-transformed gene copy number across 858 cancer cell lines from the DepMap portal. Genes meeting the significance criteria (correlation > 0.1, FDR < 5%) are highlighted. **B** Donut charts showing the chromosomal distribution of genes positively correlated with CFLAR dependency at increasing correlation thresholds of Cor > 0.10 (left), > 0.15 (middle), and > 0.17 (right). **C** Immunoblot analysis of 8p-neutral and 8p-loss cancer cell lines. Two pairs were used: breast cancer (HCC1419, 8p-neutral; HCC70, 8p-loss) and ovarian cancer (OVK-18, 8p-neutral; JHOS-4, 8p-loss). **D**, **E** Time-lapse monitoring of YOYO-3–positive cell death in the breast **D** and ovarian **E** cancer cell pairs following 1 µg/ml Dox treatment. Representative images at the 96 h-time point of i-shCFLAR are shown on the right. Scale bar represents 150 μm. **F**, **G** Immunoblot analysis of the breast **F** and ovarian **G** cancer cells treated with or without Dox for 72 h. All values in the graphs are mean ± SD (*n* = 3 from three independent experiments).

**Fig. 6:**
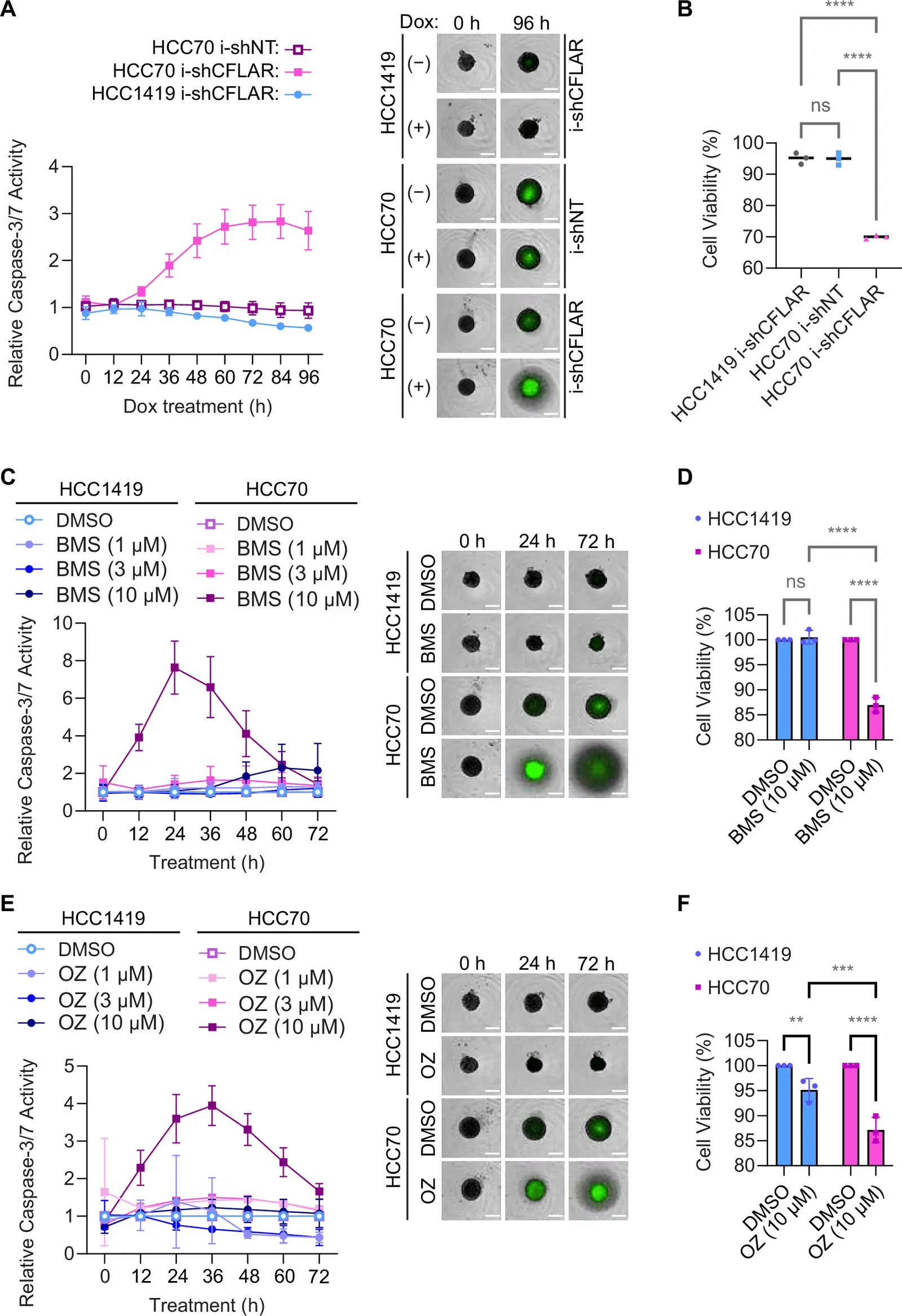
Targeting the MAP3K7–NF-κB–CFLAR axis selectively induces apoptosis in 8p-deleted tumor spheroids. **A** Time-lapse monitoring of CASP3/7 activation in i-shCFLAR–transduced HCC1419 (8p-neutral) and HCC70 (8p-loss) tumor spheroids following 1 µg/ml Dox treatment. Representative images at the indicated time points are shown on the right. Scale bar represents 400 μm. **B** Bar graph showing cell viability in tumor spheroids 72 h after treatment with Dox. Values in Dox-treated groups are normalized to the respective untreated controls. Statistical significance was determined by one-way ANOVA followed by Tukey’s multiple comparisons test. Individual data points are shown as dots; values are presented as mean ± SD. ****, *P* ≤ 0.0001; ns, not significant. **C**, **E** Time-lapse monitoring of active CASP3/7–positive cells in tumor spheroids following treatment with the indicated doses of BMS-345541 (BMS) **C** or 5Z-7-oxozeaenol (OZ) **E**. Representative images at the indicated time points for cells treated with 10 μM of each inhibitor are shown on the right. Scale bar represents 400 μm. **D**, **F** Bar graph showing cell viability in tumor spheroids 24 h after treatment with 10 µM BMS or 10 µM OZ. Values in inhibitor-treated groups are normalized to the respective DMSO-treated controls. Statistical significance was determined by two-way ANOVA followed by Tukey’s multiple comparisons test. Individual data points are shown as dots; values are presented as mean ± SD. ****, *P* ≤ 0.0001; ***, *P* ≤ 0.001; **, *P* ≤ 0.01; ns, not significant.

To determine whether CFLAR vulnerability extends to cancers with 8p loss, we next analyzed DepMap CRISPR dependency and copy number data across cancer cell lines. As expected, CASP8 (2q33.1) exhibited one of the strongest negative correlations with CFLAR dependency, whereas several apoptosis suppressor genes, including BCL2 (18q21.33), BCL2L13/BCL-RAMBO (22q11.21) and CLU (8p21.1), along with NF-κB pathway genes, including key upstream activators such as MALT1 (18q21.32), TNFRSF1B (1p36.22), and TNFRSF11A (18q21.33), and IL17RA (22q11.1) showed positive correlations (Fig. 5A). Interestingly, in addition to VPS37A, genes involved in phagophore closure, including VPS4B (18q21.33) [8], EPG5 (18q21.1) [7], CHMP7 (8p21.3) [7] and GABARAP (17p13.1) [36] were also positively correlated. Among the 1491 positively correlated genes (Cor > 0.10, q < 0.05), chromosome 1p accounted for the largest fraction (594 genes, 39.8%), followed by 17p (245 genes, 16.4%), 8p (218 genes, 14.6%), 22q (156 genes, 10.5%), 18q (110 genes, 7.4%) and 19p (100 genes, 6.7%) (Fig. 5B). Notably, the proportion of genes located on chromosome 8p increased markedly with more stringent correlation thresholds, reaching 48.9% (163 genes) at Cor > 0.15 and 82.3% (107 genes) at Cor > 0.17, suggesting that cancer cells with 8p loss exhibit increased dependency on CFLAR for survival. To test this, we analyzed chromosome copy number data from the DepMap portal and selected two sets of breast and ovarian cancer cell lines with varying 8p status: breast cancer (HCC1419, 8p neutral; HCC70, 8p loss) and ovarian cancer (OVK-18, VPS37A 8p-neutral; JHOS-4, 8p-loss). After validation of VPS37A and TNFRSF10B/DR5 reductions in 8p-loss cells (Fig. 5C), the cells were transduced with Dox-inducible CFLAR shRNAs and monitored for cell death upon Dox exposure. CFLAR depletion killed 8p-loss, but not 8p-neutral, cells, accompanied by cleavage of CASP8, CASP3, and PARP (Fig. 5D–G), indicating that apoptosis was selectively induced in 8p-loss cells. However, restoration of VPS37A alone failed to rescue CFLAR dependency in 8p-loss cancer cells (Fig. S3A–D), in contrast to VPS37A knockout cells (Fig. 3A, B**)**, suggesting that the observed vulnerability reflects the combined effects of multiple genes within the 8p region. We next evaluated the effects of CFLAR depletion in 3D spheroid models of 8p-loss and 8p-neutral cancer cells. Since the ovarian cancer cell lines failed to form spheroids, the analysis focused on breast cancer cells. Consistently, we found that CFLAR depletion more robustly activated CASP3/7 and decreased cell viability in 8p-loss tumor spheroids, relative to 8p-neutral spheroids (Fig. 6A, B). Similar effects were observed upon treatment with JSH-23 (Fig. S4A, B) as well as the MAP3K7 inhibitor 5Z-7-oxozeaenol [37] and the IKK inhibitor BMS-345541 [38] (Fig. 6C–F). Collectively, these results suggest that targeting the MAP3K7–NF-κB–CFLAR axis can selectively induce apoptosis and suppress tumor growth in 8p-deleted cancer cells.

## Discussion

Recurrent loss of chromosome 8p is observed across multiple human cancers and is associated with poor prognosis [19–21]. VPS37A, a gene located on chromosome 8p, encodes an ESCRT-I component involved in endosomal receptor sorting [39] and phagophore closure [15]. We recently found that VPS37A deficiency primes cells for apoptosis by stabilizing CASP8 assembly on ATG8ylated phagophores [11]. Here, we identify the MAP3K7–NF-κB–CFLAR axis as a synthetic vulnerability in VPS37A-deficient cells, where its inhibition selectively activates CASP8-mediated apoptosis and suppresses tumor growth. Although VPS37A loss often coincides with deletion of TNFRSF10A and TNFRSF10B, key death receptors on 8p that initiate CASP8 activation via the extrinsic apoptotic pathway [40], targeting this axis still efficiently induces apoptosis in 8p-deleted cancer cells. Notably, our data show that ectopic expression of VPS37A alone is insufficient to rescue CFLAR dependency in 8p-loss cancer cells, suggesting that this dependency is likely driven by the combined effects of multiple 8p genes in addition to VPS37A. CHMP7 and CLU, which have been implicated in phagophore closure [7] and inhibition of pro-apoptotic BAX [41], respectively, are potential contributors that could stabilize CASP8 assemblies and promote their full activation via the mitochondrial amplification loop. Further studies are warranted to identify additional factors underlying susceptibility to CASP8-mediated apoptosis in 8p-loss cancer cells. Nonetheless, since VPS37A/8p deletion arises early in tumorigenesis and persists throughout progression, these findings reveal a CASP8-dependent vulnerability that may be therapeutically exploitable in cancers harboring 8p loss.

In addition to promoting non-canonical CASP8 activation at phagophores, disruption of ESCRT-I can induce cell death by causing endosomal accumulation and ligand-independent activation of cytokine receptors [12, 31]. Using two distinct VPS37A mutants with reciprocal defects in autophagy and endosomal receptor sorting [15, 18], we show that impaired phagophore closure is the primary driver of the selective vulnerability to CFLAR depletion in VPS37A-deficient cells. Consistently, ATG5 depletion, which blocks ATG conjugation required for CASP8 recruitment to the phagophore [42], abolishes this vulnerability. These observations agree with the fact that VPS37A is uniquely required for phagophore closure, whereas its role in endosomal sorting can be largely compensated by VPS37B-D [13–15]. Co-targeting these paralogues together with CFLAR may enable simultaneous activation of pro-death signaling from perturbed endosomal and autophagosomal membranes, thereby amplifying cell death in 8p-deleted cancers.

The observation that VPS37A copy number loss correlates with MYC copy number gain is consistent with previous studies indicating that 8p loss and 8q gain represent commonly linked genomic alterations in human cancers [19, 20, 30]. Interestingly, MYC can repress the expression of autophagy and lysosomal genes via competing with the transcriptional factors TFEB and TFE3 [43]. Moreover, CFLAR loss has been shown to compromise autophagic flux via destabilizing BECN1, a component of the class III phosphatidylinositol-3-kinase complex [44]. Given that 8p deletion sensitizes non-malignant MCF10A mammary epithelial cells to the lysosome inhibitor chloroquine [20], downregulation of upstream autophagy factors by CFLAR depletion, together with MYC amplification, may synergize with VPS37A loss to impair autophagic flux and thereby suppress the growth of 8p-deleted cancers, independent of CASP8 activation.

Recent studies have shown that VPS37A mediates lysosomal degradation of STING vesicles, thereby terminating innate immune signaling [45, 46]. Impaired autophagy-dependent mitochondrial clearance can further enhance STING signaling by increasing cytosolic mtDNA [47, 48]. STING activation promotes IRF3-dependent transcription of type I interferons and enhances anti-tumor immunity [49, 50]. Although no correlation was found between VPS37A copy number and STING1–IRF3 dependencies, hyperactivation of STING can autonomously induce cell death through its channel activity, bypassing IRF3 [51]. However, STING signaling also activates NF-κB, which upregulates inflammatory cytokines and immune checkpoint molecules, paradoxically promoting tumor growth, metastasis, and immune evasion [49, 50]. The in vivo experiments in this study utilized STING1-negative U-2 OS cells [52] in immunodeficient mice, limiting assessment of the interplay between CASP8-dependent vulnerability in 8p-deleted cancer cells and STING signaling. Given that CFLAR vulnerability in 8p-deleted cells can be pharmacologically targeted via the MAP3K7–NF-κB axis, future studies are warranted to determine whether combining NF-κB inhibition with STING activation could selectively induce apoptosis while enhancing anti-tumor immunity in 8p-deleted tumors.

In addition to chromosome 8p, our analysis revealed that genes located on other chromosomal loci, including 1p, 17p, 22q, 18q, and 19p, were enriched among genes whose copy number positively correlates with CFLAR dependency. The functional relevance of these loci to CFLAR dependency remains to be defined. Interestingly, chromosome 18q, which is also frequently deleted across multiple cancer types, harbors VPS4B, whose loss has been shown to confer VPS4A dependency through a synthetic lethal interaction [53, 54]. Chromosome 18q also encodes anti-apoptotic BCL2 as well as EPG5, which is required for autophagic flux [55] and has been suggested to be involved in phagophore closure [15]. Given the critical roles of CFLAR in regulating non-canonical CASP8 activation and autophagic flux, it is conceivable that the combined loss of these genes may similarly sensitize cancer cells to CFLAR inhibition or CASP8-dependent apoptosis. Thus, CFLAR-associated vulnerabilities may extend beyond 8p to other recurrent chromosomal deletions, warranting further investigation.

## Methods

### Reagents

The following antibodies were used for immunofluorescence (IF) and immunoblotting (IB): mouse antibodies against ACTB/β-ACTIN (Sigma-Aldrich, A5441; IB, 1:10,000); rabbit antibodies against cleaved CASP3 (Cell Signaling Technology, 9661; IB, 1:1,000), cleaved CASP8 (Cell Signaling Technology, 9496; IB, 1:1,000), pro CASP8 (abcam, ab108333; IB, 1:1,000), CFLAR/FLIP (Cell Signaling Technology, 56343; IB, 1:1,000), GFP (Cell Signaling Technology, 2956; IB, 1:2,000), MAP1LC3B (Novus, NB100-2220; IB, 1:5,000), PARP (Cell Signaling Technology, 9542; IB, 1:1,000), TNFRSF10B/DR5 (Cell Signaling Technology, 8074; IB, 1:1,000), VPS37A (Sigma-Aldrich, HPA024705; IB, 1:4000); guinea pig antibodies against SQSTM1/p62 (American Research Products, 03-GP62-C; IB, 1:4,000). SMART Pool Non-targeting (D-001810-10) and Human CFLAR (L-003772-00-0005) siRNAs were purchased from Horizon Discovery. To generate pRSITEP-U6Tet-sh human CFLAR-EF1-TetRep-2A-Puro, target sequence of human CFLAR was designed using VectorBuilder (Table S4). These oligos were annealed and phosphorylated then ligated with pRSITEP-U6Tet-sh-EF1-TetRep-2A-Puro (CELLECTA, SVSHU6TEP-L, linearized). In order to generate pRSITEP-U6Tet-sh non-targeting-EF1-TetRep-2A-Puro, the target sequence was referred from scramble shRNA (1864, Addgene). These oligos (Table S4) were annealed and phosphorylated then ligated with pRSITEP-U6Tet-sh-EF1-TetRep-2A-Puro. epiCRISPR (135960) was obtained from Yongming Wang through Addgene. For CRISPR-Cas9-mediated gene silencing, each gRNA listed in Table S4 was subcloned into the Sap1 site of epiCRISPR to generate VPS37A KO cells, ATG5/VPS37A and CASP8/VPS37A DKO cells. pCDH1-CMV-GFP-FL(VPS37A variant 1)-EF1-Puro and pCDH1-CMV-GFP-ΔUEVL(VPS37A variant 4)-EF1-Puro were previously generated [15]. The VPS37A point mutation variant (K382D) cDNA was generated using Gibson Assembly. Briefly, to generate pCDH1-CMV-GFP-K382D-EF1-Puro, oligo1 5’-ACACAGATGAAGTCCACTTTCGAAAAGAAGATGCAAAGG-3’ and oligo2 5’-GCTGAAGGTCCTCTTCCTTGGCTCTTCTAC-3’ were annealed for fragment1, also oligo3 5’-CAAGGAAGAGGACCTTCAGCAGGCGATAGC-3’ and oligo4 5’-CTGACGGGCACCGGAGCGATCGCAGATCCTTGCGGCCG-3’ were annealed for fragment2. These two fragments were assembled into the BstBI–BamHI site of pCDH1-CMV-GFP-FL(VPS37A variant 1)-EF1-Puro [15] by Gibson Assembly. All other reagents were obtained from the following sources: BMS-345541 (Sigma-Aldrich, B9935); bovine serum albumin (BSA; EMD Millipore, 126575); Caspase-3/7 Apoptosis Assay Reagent (Essen Biosciences, 4704); Caspase-Glo 8 Assay kit (Promega, G8200); CellEvent Caspase-3/7 Green Detection Reagent (Invitrogen, C10423); Cytotox Dye for Counting Dead Cells (SARTORIUS, 4846); Dox hyclate (Sigma-Aldrich, D9891); dimethyl sulfoxide (DMSO; Sigma-Aldrich, D2438); Dulbecco’s Modification of Eagle’s Medium (DMEM; CORNING, 10-013-CV); Incucyte Caspase-3/7 Red Dye (Essen Biosciences, 4704); jetPRIME (Polyplus, 101000015); 5Z-7-oxozeaenol (MedChemExpress, HY-12686); Phosphatase Inhibitor Cocktail 2/3 (Sigma-Aldrich, P5726/ P0044); Protease Inhibitor Cocktail (Sigma-Aldrich, P8340); YOYO-3 iodide (Thermo Fisher Scientific, Y3606).

### TCGA and DepMap datasets and gene enrichment analyses

Gene copy number and mRNA expression data from The Cancer Genome Atlas Program (TCGA) patient cohorts were obtained from cBioPortal [56–58] (https://www.cbioportal.org/). CRISPR gene dependency data across cancer cell lines (Public 25Q2, Chronos scores) were analyzed using the DepMap portal (https://depmap.org/). For gene enrichment studies, Reactome pathway [59] and transcription factor RegNetwork [60] analyses were performed using Metascape [61] (https://metascape.org/) and ShinyGO [62] (https://bioinformatics.sdstate.edu/go/), respectively.

### Cell culture, transfection and viral transduction

U-2 OS cells (ATCC, HTB-96) were maintained at 37°C and 5% CO2 in McCoy’s 5 A Medium supplemented with 10% fetal bovine serum (FBS) and 1× Antibiotic Antimycotic Solution (AA; Corning, 30–004-CI). OVK18 cells (RIKEN BioResource Center) and 293 T/17 cells (ATCC, CRL-11268) were cultured in DMEM supplemented with 10% FBS and 1× AA. HCC70, HCC1419 cells (ATCC, CRL-2315) were cultured in RPMI-1640 supplemented with 10% FBS and 1× AA. JHOS-4 (RIKEN BioResource Center) cells were cultured in DMEM/F-12 50/50 supplemented with 10% FBS and 1× AA. For siRNA-mediated gene silencing, the indicated siRNAs were transfected into cells using Lipofectamine RNAiMAX transfection reagent according to the manufacturer’s protocol. U-2 OS-X cells were generated through two rounds of transplantation in NRG mice as described below. VPS37A KO, ATG5 KO, CASP8 KO, ATG5/VPS37A DKO, and CASP8/VPS37A DKO U-2 OS-X cells were generated by transfection with the respective gRNA-encoded epiCRISPR vectors using the jetPRIME transfection reagent for 2 days, followed by selection with 2 µg/ml puromycin for 10 days. Five single clones were pooled and used for experiments. Recombinant lentiviruses were produced in 293 T/17 cells and infected into target cells as described previously [15], to transduce GFP-tagged wild-type or mutant VPS37A, GFP alone, Dox-inducible CFLAR (i-shCFLAR), or non-targeting (i-shNT) shRNAs. VPS37A KO U-2 OS-X1 cells stably transduced with i-shCFLAR were generated as described below.

### Generation of transplantable U-2 OS sublines and in vivo assessment of CFLAR depletion

To establish efficiently transplantable U-2 OS sublines, U-2 OS cells suspended in 50% Matrigel Membrane Matrix at a concentration of 4 × 10⁷ cells/ml were injected paratibially (1 × 10⁶ cells in 25 µl) into 6–8-week-old NRG mice (Jackson Laboratory, 007799). Seventeen weeks after injection, cells from tumors that had formed were harvested and re-injected into mice under the same conditions. Seven weeks following re-injection, harvested cells were established as U-2 OS-X cells. VPS37A KO U-2 OS-X1 cells stably transduced with i-shCFLAR were established from i-shCFLAR/VPS37A KO U-2 OS-X tumors. To assess the effect of CFLAR depletion on osteosarcoma progression, i-shCFLAR/WT U-2 OS-X cells and i-shCFLAR/VPS37A KO U-2 OS-X1 cells were injected paratibially as described above. Three weeks after injection, mice in the treatment groups began receiving 200 ppm Dox in the diet, which continued for the duration of the experiment. Tumor volumes were measured weekly and calculated as πLW^2^/6, where L and W are the length and width measured with calipers. The humane endpoint was defined as a tumor volume of 2000 mm³.

### Immunoblotting

Total cell lysates were prepared in radio-immunoprecipitation assay buffer (150 mM NaCl, 10 mM Tris-HCl, pH 7.4, 0.1% SDS, 1% Triton X-100, 1% Deoxycholate, 5 mM EDTA, pH 8.0) containing protease (Sigma-Aldrich, P8340) and phosphatase (Sigma-Aldrich, P5726) inhibitors, and subjected to SDS-PAGE followed by immunoblotting with the indicated antibodies. The signal intensities were quantified using the Image Studio version 5 software (LI-COR).

### Cell death assays

The IncuCyte S3 and SX5 Live-Cell Analysis System (Essen BioScience; 10× objective) was used to monitor cell death induction and CASP3 or CASP7 activation as described previously [9]. Briefly, cells plated on 96-well plates were cultured overnight and incubated with 500 nM Cytotox NIR, 100 nM YOYO-3 iodide or 500 nM IncuCyte Caspase-3/7 Red Dye in the presence or absence of the indicated compounds. Fluorescence and phase images were then obtained at the indicated intervals and analyzed using the Basic Analyzer or Cell-by-Cell Analysis (Essen BioScience, 9600-0031) modules of the IncuCyte S3 Software (Essen BioScience, Version 2024 A) and the IncuCyte SX5 Software (Essen BioScience, Version 2022B).

### Tumor spheroid assay

HCC70 and HCC1419 cells were seeded at a density of 3,000 cells in 100 μL of growth medium in 3D culture Ultra-low Attachment (ULA) Plates: 96 well, U bottom, White plates (S-bio, MS-9096WZ, for CellTiter-Glo 3D) and ULA Plates: 96 well, U bottom, Clear plates (S-bio, MS-9096UZ, for IncuCyte spheroid imaging). After 3 days of seeding, the formed spheroids were exposed with indicated compounds in indicated duration, and their ATP levels were measured using CellTiter-Glo 3D Cell Viability Assay (Promega, G9683) after 24 h exposure of indicated treatments, also the spheroid formation and caspase-3/7 activation were assessed by using IncuCyte Spheroid Analysis Software Module (SARTORIUS, 9600-0019) to monitor at every 6 h for 72–96 h after starting indicated treatments.

### Statistical analyses

Statistical significance was determined using GraphPad Prism 7.0. Threshold for statistical significance for each test was set at 95% confidence (*p* < 0.05). All data from in vitro studies are representative of three independent experiments. For animal studies, the sample size was determined using a two-sample t-test analysis at the test power of 0.80, set significance level α = 0.05, and an effect size based on preliminary experiments.

## Supplementary information

SUPPLEMENTAL MATERIALS

S Tables

## Data Availability

Data are available upon request.
