# Supplementary material for: VPS37A loss creates CASP8-dependent vulnerability via the MAP3K7–NF-κB–CFLAR axis
Source: Cancer Gene Ther. 2026 Jul 7;33(8):897–908. doi: 10.1038/s41417-026-01054-3 (PMC13498462; doi:10.1038/s41417-026-01054-3)
Supplement: Supplementary file 1 [file 41417_2026_1054_MOESM1_ESM.docx]

**Supplementary Materials for**

**VPS37A loss creates CASP8-dependent vulnerability via the MAP3K7–NF-κB–CFLAR axis**

Tatsuya Hattori^1,2^, Longgui Chen^1^, Xinwen Liang^1^, Kouta Hamamoto^1^, Hong-Gang Wang^1,2^ and Yoshinori Takahashi^1,2^

^1^Division of Pediatric Hematology and Oncology, Department of Pediatrics, The Pennsylvania State University College of Medicine, Hershey, PA 17033, USA; ^2^Department of Cell and Biological Systems, The Pennsylvania State University College of Medicine, Hershey, PA 17033, USA

Correspondence to Yoshinori Takahashi: [ytakahashi@pennstatehealth.psu.edu](mailto:ytakahashi@pennstatehealth.psu.edu) or Hong-Gang Wang: [huw11@psu.edu](mailto:huw11@psu.edu)

**Figure S1. VPS37A loss sensitizes U-2 OS cells to the NF-κB inhibitor JSH-23 in U-2 OS cells.** Time-lapse monitoring of Cytotox-positive dead U-2 OS cells following treatment with the indicated concentrations of JSH-23 using the IncuCyte Live-Cell Analysis System.

**Figure S2. VPS37A loss sensitizes U-2 OS-X cells to NF-κB inhibition and CFLAR depletion.** (**A**) Time-lapse monitoring of Cytotox-positive dead U-2 OS-X cells following treatment with the indicated concentrations of JSH-23 using the IncuCyte Live-Cell Analysis System. (**B**) Time-lapse monitoring of YOYO-3–positive dead U-2 OS-X cells expressing inducible CFLAR shRNA (i-shCFLAR) following treatment with 1 µg/ml doxycycline (Dox).

**Figure S3. VPS37A overexpression is insufficient to mitigate sensitivity to CFLAR loss in 8p-deleted cancer cells.** (**A, B**) Time-lapse monitoring of Cytotox-positive dead HCC70 (**A**) and JHOS-4 (**B**) cells expressing inducible non-targeting (i-shNT) or CFLAR (i-shCFLAR) shRNA following treatment with 1 µg/ml doxycycline (Dox). Representative images at 96 h (HCC70) and 72 h (JHOS-4) after Dox treatment are shown on the right. Scale bar, 150 μm. (**C, D**) Immunoblot analysis of HCC70 (**C**) and JHOS-4 (**D**) cells treated with 1 µg/ml Dox for 24 hours. All values are mean ± SD (= 3 from three independent experiments).

**Figure S4. 8p-deleted HCC70 spheroids show increased sensitivity to NF-κB inhibition relative to 8p-neutral HCC1419 spheroids**. (**A**) Time-lapse monitoring of CASP3/7 activation in i-shCFLAR–transduced HCC1419 (8p-neutral) and HCC70 (8p-loss) tumor spheroids following treatment with the indicated concentrations of JSH-23. Representative images at 72 h after treatment are shown on the right. Scale bar, 400 μm. (**B**) Immunoblot analysis verifying depletion of c-FLIP proteins following treatment with JSH-23 (80 µM, 16 h).

**Table S1. Abbreviations of TCGA tumor types**

**Table S2. Top 10 Reactome pathways enriched among VPS37A codependent genes**

**Table S3. Top 10 transcription factors predicted to regulate VPS37A synthetic lethal partner candidates**

**Table S4. Oligonucleotides used for plasmid construction**
